# Can delusions be understood linguistically?

**DOI:** 10.1080/13546805.2016.1190703

**Published:** 2016-06-20

**Authors:** Wolfram Hinzen, Joana Rosselló, Peter McKenna

**Affiliations:** ^a^ Catalan Institute for Advanced Studies and Research (ICREA), Departament de Traducció i Ciències del Llenguatge, Universitat Pompeu Fabra, Barcelona, Spain; ^b^ Department of Linguistics, University of Barcelona, Barcelona, Spain; ^c^ FIDMAG Germanes Hospitalàries Research Foundation, Barcelona, Spain; ^d^ CIBERSAM, Barcelona, Spain; ^e^ Department of Philosophy, Durham University, Durham, UK

**Keywords:** Schizophrenia, cognition, language, delusions

## Abstract

Delusions are widely believed to reflect disturbed cognitive function, but the nature of this remains elusive. The “un-Cartesian” cognitive-linguistic hypothesis maintains (a) that there is no thought separate from language, that is, there is no distinct mental space removed from language where “thinking” takes place; and (b) that a somewhat broadened concept of grammar is responsible for bestowing meaning on propositions, and this among other things gives them their quality of being true or false. It is argued that a loss of propositional meaning explains why delusions are false, impossible and sometimes fantastic. A closely related abnormality, failure of linguistic embedding, can additionally account for why delusions are held with fixed conviction and are not adequately justified by the patient. The un-Cartesian linguistic approach to delusions has points of contact with Frith’s theory that inability to form meta-representations underlies a range of schizophrenic symptoms. It may also be relevant to the nature of the “second factor” in monothematic delusions in neurological disease. Finally, it can inform the current debate about whether or not delusions really are beliefs.

Delusions, as seen especially in schizophrenia, but also in mania, depression and certain types of organic brain disease, are arguably the least understood of all psychotic symptoms. Over the years, theories have ranged from the biological, as in the currently highly influential aberrant salience account (Corlett, Taylor, Wang, Fletcher, & Krystal, 2010; Fletcher & Frith, 2009; Kapur, 2003), all the way to the philosophical (Bortolotti, 2010; Currie, 2000; Radden, 2011; Sass, 1995). However, it is fair to say that the majority of theoretical approaches have been psychological in nature, that is they propose that changes in one or more aspects of higher cognitive function give rise to delusions or at least lay the groundwork for them to appear.

As we argue later, the main current examples of this last group of theories face important limitations, either theoretically or in terms of their experimental support. In these circumstances, it seems reasonable to propose a new approach, one that revolves around a novel conceptualisation of how language relates to thought. The “un-Cartesian” hypothesis of Hinzen and co-workers (Arsenijevic & Hinzen, 2012; Hinzen, 2006, 2007, 2012, 2015; Hinzen & Sheehan, 2013; Sheehan & Hinzen, 2011) essentially maintains that there is no distinct mental space removed from language in which thinking takes place. Also, according to this theory a somewhat broadened concept of grammar is responsible for bestowing on utterances sentence level or “propositional” meaning, the kind of meaning that arises from subject–predicate combinations and which among other things gives assertions their quality of being true or false. Since the defining characteristic of delusions is their erroneous and excessive truth value, taking an un-Cartesian perspective could have explanatory value.

## Delusions: what needs to be explained

Delusions, as they are defined in numerous psychiatric textbooks, and also in the major disease classification systems of DSM 5 and ICD-10, are false beliefs that are held with fixed conviction*,* in the face of what is usually obvious evidence to the contrary, and which are not susceptible to counter-argument. Cultural or shared minority beliefs are typically excluded. The themes that delusions take vary widely from patient to patient, and several different types of delusion may also be present in a single patient – such “polythematic” delusions are perhaps most typically seen in schizophrenia. The most detailed classification of delusions according to their content is that in the Present State Examination (PSE) (Wing, Cooper, & Sartorius, 1974), a structured psychiatric interview that has the benefit of a great deal of richness and psychopathological sophistication. The main types of delusion according to this scheme are outlined in Table 1.

**Table 1.** The PSE classification of delusions (from Wing et al., 1974).

*Delusions of control*
The subject feels under the control of some force or power other than himself, for example, which makes his movements for him without him willing it, or uses his voice or his handwriting, or replaces his personality.
*Delusional mood*
The subject feels that his familiar environment has changed in a way which puzzles him and which he may not be able to describe clearly. Everything feels odd, strange and uncanny, something suspicious is afoot, events are charged with new meaning.
*Delusions of reference*
People drop hints about what the subject says, or says things with a double meaning, or do things in a special way so as to convey a special meaning. The whole neighbourhood may seem to be gossiping about the him, far beyond the bounds of possibility, or he may see references to himself on the television or in newspapers. He may seem to be followed, his movements observed, and that what he says tape-recorded.
*Delusions of misinterpretation and misidentification*
A further extension of the delusion of reference in which situations appear to be created which have a special meaning. Things seem to be specially arranged to test the patient out, objects are arranged so that they have a special significance for him, street signs or advertisements on buses or patterns of colour seem to have been put there in order to give him a message.
*Delusions of persecution*
Someone is deliberately trying to harm him, for example, poison him or kill him.
*Delusions of assistance*
The subject believes the same forces, powers and organisations are endeavouring to help him in surreptitious ways – to direct his life, to enable him to become a better person and so on.
*Delusions of grandiose abilities*
The subject believes he has special abilities or powers, for example, he is much cleverer than anyone else, has invented machines, composed music or solved mathematical problems, etc., beyond most people’s comprehension, or there is a special purpose or mission to his life.
*Delusions of grandiose identity*
The subject believes he is famous, rich, titled or related to prominent people.
*Religious delusions*
The subject believes he is specially close to Christ or God, is a saint, has special spiritual powers, etc.
*Delusional explanations in terms of paranormal phenomena*
The subject is influenced by hypnotism, telepathy, or the occult.
*Delusional explanations in terms of physical forces*
Electricity, X-rays, radio-waves or similar are affecting the subject
*Delusions of alien forces penetrating or controlling mind (or body)*
Any delusion which involves an external force penetrating the subject’s mind or body, for example, rays turn his liver to gold, alien thoughts pierce his skull or are inserted into his mind, hypnotism makes him levitate.
*Primary delusions*^a^
Delusions where a subject suddenly becomes convinced that a particular set of events has a special meaning.
*Sexual delusions*
Any delusion with sexual content, for example, fantasy lover, sex changing, etc.
*Delusions of guilt*
The subject feels he has committed a crime, or sinned greatly, or deserves punishment.
*Simple delusions concerning appearance*
The subjects nose is too large, teeth misshapen, body crooked, etc.
*Delusions of depersonalisation*
Subject has no head, does not exist, hollow instead of a brain, etc.
*Hypochondriacal delusions*
Subject has incurable cancer, bowels are stopped up, insides are rotting, etc.
*Delusions of catastrophe*
The subject believes that the world is about to end, some catastrophe has happened or will occur, or everything is evil and will be destroyed.
*Morbid jealousy*
The subject believes his partner is being unfaithful.

^a^This rare type of delusion is also known as delusional perception.

Table 1 also suggests a number of more conceptually based subgroupings of delusions. One obvious distinction that can be made is between delusions where the central feature is attribution of personal significance to neutral events – delusional mood, delusions of reference and delusions of misinterpretation – and those where the patient simply makes a false assertion and no component of attribution of significance is apparent. Delusions of the former type are customarily referred to as referential delusions. There is no collective term for the latter type, apart from the now somewhat quaint-sounding term used by Schneider (1949/1974), delusional intuitions. We will use the term *propositional delusions* for these delusions, as a more modern and neutral alternative to Schneider’s term.^1^

Within the category of propositional delusions, there also seems to be a distinction between beliefs that are simple erroneous assertions that something is true, and those where the belief, while being demonstrably false, held in the face of counter-argument, and so on, also has an obvious link to another form of psychopathology. Examples here would include a belief that the auditory hallucinations a patient is experiencing are due to there being a radio transmitter in his or her head (delusional explanations in the PSE), or wrongly stating that one has committed a crime in the context of major depression (one of several categories of mood-congruent delusion). In the same vein, so-called nihilistic delusions, where one believes one has no brain, is empty, does not exist, etc., are widely considered to be a delusional elaboration of another symptom, depersonalisation (Sierra, 2009).

Another important feature of delusions is the varying degrees of improbability that they show. Almost all delusions are factually highly unlikely, for example that there is a conspiracy against the person involving the Masons or the Catholic church (or both). But often delusions go beyond this into the realm of the bizarre – a well-known example here is the mathematician John Nash’s belief that he was shortly going to be made emperor of Antarctica by aliens. The most extreme manifestation of this tendency is so-called fantastic delusions, beliefs that violate common sense at its most elementary, as in patients who say that they have travelled to other planets, have had thousands of children, or that Prince Charles and Lady Diana were present at their birth (even though these Royals would themselves have been children at the time).

A final, somewhat neglected feature of delusions is the way in which they are justified, or rather not justified. Attempts to get patients to explain their reasons for holding a delusional belief invariably go nowhere – a not-infrequent response is that they simply know it is true and no further elaboration is necessary. When an explanation is provided, it is typically illogical or absurd. Thus, a patient might say he believes he is a rock star who has had his memories of this part of his life removed because he has brief flashes of memory of himself performing before thousands of people, something that has other, more plausible explanations; or he might offer as evidence for the belief he is being forced to engage in sexual activities when he is asleep the observation that when he wakes up the position of his shoelaces is different from when he went to bed.

## Theories of delusions

For a considerable portion of the twentieth century, theories of delusions did not stray far from the descriptive. Particularly influential in this era were the views of the psychiatrist and philosopher Jaspers (1959; see also Walker, 1991), who systematically attempted to capture the nature of abnormal subjective experiences using what he called “phenomenology”,^2^ that is, in and of themselves, and without reference to extra psychological (or physical) theorising. Using this method, he was able to deduce a certain amount about the nature of delusions, most famously arguing that they were psychologically irreducible and did not emerge comprehensibly from anything in the individual’s personality or life experiences.

Going some way beyond his own phenomenological remit, Jaspers went on to argue that the nature of the delusional experience fundamentally involved awareness of meaning, which underwent a radical transformation: “There is an immediate, intrusive attribution of the meaning … and it is this which is itself the delusional experience.” This view, however, was not shared by another phenomenologically minded psychiatrist of the day, Schneider (1949/1974). He took the position that only referential delusions could be understood in this way. As he legitimately pointed out, patients with what he called delusional intuitions, which we have renamed for convenience propositional delusions, held beliefs – about a summons from God, special powers, of persecution, of being loved, etc. – that simply occurred to them, and in which no attribution of abnormal significance was discernible. Often these beliefs seemed to arise de novo and so it was also difficult to argue that abnormal significance had previously been a factor in their development.

More recently there have been several attempts to explain delusions psychologically. One of the most important of these has been Frith’s (1992) proposal that theory of mind impairment, that is, an inability to ascribe mental states to other people, leads deluded patients to attribute faulty intentions to others’ actions. This could then account for referential and persecutory delusions – patients with the former incorrectly interpret other people’s words and body language as attempts to communicate obliquely with them, and those with the latter cannot read people’s intentions properly and so fall prey to ideas that others intend them harm. At first sight this theory appears to have impressive experimental support: it is well established that patients with schizophrenia show impaired performance on theory of mind tasks, and that the degree of impairment is substantial and disproportionate to the general level of cognitive impairment seen in the disorder (Bora, Yucel, & Pantelis, 2009; Sprong, Schothorst, Vos, Hox, & van Engeland, 2007). Unfortunately, it has also become clear that theory of mind impairment is not associated with presence and severity of the “reality distortion” syndrome (i.e., delusions and hallucinations) (Ventura, Wood, & Hellemann, 2013), and most studies do not find evidence for a relationship with delusions specifically (Abdel-Hamid et al., 2009; Drury, Robinson, & Birchwood, 1998; Greig, Bryson, & Bell, 2004; Walston, Blennerhassett, & Charlton, 2000), something that clearly ought to be the case if the theory were correct.

The other currently influential cognitive theory of delusions is probabilistic reasoning bias, or “jumping to conclusions”. This theory is based on an experimental paradigm where subjects are shown a series of beads which they are informed are being drawn from one of two concealed jars containing contrasting proportions of beads of a particular colour (in a typical design one jar contains 85 black and 15 white beads and the other contains 15 black and 85 white beads). The subjects have to indicate when they have reached a firm conclusion about which of the jars the beads are being drawn from. In the original study by Huq, Garety, and Hemsley (1988), schizophrenic patients were found to require significantly less draws to decision than healthy controls. Many subsequent studies have confirmed this finding (for a review see Garety & Freeman, 2013). However, in much the same way as with theory of mind impairment, these studies have struggled to demonstrate an association with presence or severity of delusions (Dudley et al., 2011; Falcone et al., 2015; Freeman et al., 2014; Garety et al., 2013; Langdon, Ward, & Coltheart, 2010; Lincoln, Ziegler, Mehl, & Rief, 2010; Menon, Pomarol-Clotet, McKenna, & McCarthy, 2006; Moritz & Woodward, 2005; Mortimer et al., 1996; Ochoa et al., 2014; Peters, Thornton, Siksou, Linney, & MacCabe, 2008; So et al., 2012).

In summary, although it seems hard to believe that delusions do not involve some kind of alteration in cognitive processes underlying thinking, this has not proved easy to pin down. Could it be that we are inadvertently overlooking some aspect of cognitive function because we believe it cannot be relevant to thinking? Or perhaps it is our assumptions about what thought actually is that are faulty? Both these ideas are key features of the un-Cartesian hypothesis, which is outlined in the next section.

## Introducing the un-Cartesian hypothesis

The view that language and thought are separate from each other has been central to the rationalist tradition of thought for more than 300 years, starting from “Cartesian” linguists who took thought to be rational and universal by definition, whereas language was taken to be an expressive and conventional tool, though one significantly mirroring the structures of thought. A categorical separation between language and thought was also part and parcel of the discipline of linguistics that established itself in the mid-20th century; this explicitly cautioned, for methodological and empirical reasons, against identifying the domains of language and thought with one another, preferring to treat the former autonomously. Thus, “Universal Grammar” in Chomsky’s (1965) sense is a theory of the acquisition of language viewed as the study of the “language organ”, an innate disposition for language in humans. The principles governing thought remain unaccounted for in this paradigm; they are not among its concerns.^3^

As linguists started working on the principles and parameters of the new Universal Grammar, the philosopher Fodor (1983) was consolidating the conceptual foundations of cognitive science under the banner of the modularity of mind. He explicitly denied the possibility of subjecting thought to the same kind of computational treatment that Chomsky had inaugurated for language (Fodor, 2000). He also (Fodor, 2001) continued the Cartesian theme of separating language from thought and regarding the latter as primary with respect to the former. In line with this, Fodor assumed that babies already think (in the so-called “Language of Thought”, LOT) before they talk, and that other species could also think, before one arose that could additionally *express* what it was thinking.

According to the “Cartesian” view, then, language is in effect a tool that humans have developed for purely expressive purposes. It “externalises” thought – but like a printer, which also “externalises” information processed in the computer to which it is attached via an interface, it does not determine the contents of our thoughts. Thought, in contrast, is something different, somehow lying “behind” language and operating according to different principles. The un-Cartesian hypothesis (Arsenijevic & Hinzen, 2012; Hinzen, 2006, 2007, 2012, 2015; Hinzen & Sheehan, 2013; Sheehan & Hinzen, 2011) challenges this notion. It proposes that there is no separate conscious thought that is subsequently expressed in language, and what we call thought, insofar as it is *sapiens*-specific and conscious, is ultimately linguistic in nature.

This is not to say that human language developed in a vacuum. Both animals and babies filter, analyse and classify incoming sensory information in a process whereby significances for behaviour form. As a result of this, what can be referred to as *meaningful categories* develop; these can be quite complex and abstract and are often only obscurely related to their immediate perceptual features – for example, human infants are able to classify objects that may be visually quite different as “boxes”, “cars”, “toys”, “cats”, etc. (Xu, 2005; see also Carey, 2009 for a review). Since these structures are inferentially rich, forming a background for the organism’s reaction to and anticipation of stimuli, and have a content not definable in terms of physical features of the stimulus, it seems right to say that they come with a crude form of semantics: a content which does not mirror objects out there as independently described. The cognitive operations performed by these prelinguistic semantic structures, however, still represent an involuntary and online process; it awaits stimuli in their particular contexts to trigger responses and output is always contingent on relevant perceptual input.

At some point in human evolution, however, these prelinguistic semantic structures became *lexicalised*: they were given a phonological identity and became entities freely available in our minds, manipulable irrespective of the presence of any perceptual stimulus. At this point responses to experience no longer needed to be controlled by stimuli – output was no longer constrained by input – and it could become “creative” in the way Chomsky (1959) used this term in his famous criticism of Skinner.^4^

Nevertheless, even though now de-coupled from perception, stimulus-free and independently manipulable, these lexical items did not as yet constitute a language. Specifically they lacked a process to recouple them to the world on occasions of activating them or retrieving them from semantic memory. The un-Cartesian hypothesis identifies this system with grammar, or more properly a somewhat broadened concept of grammar.^5^ This provides specific rules for the manipulation of words in phrasal and sentential contexts, so that they acquire properties beyond their lexical contents. Thus, whereas MAN is a lexical item, “the man” is not (it is not listed in the lexicon), and it is only the latter, a grammatical *phrase*, which can in turn enable a fundamental process of language to take place, that of *reference*, the ability to refer to specific objects in the world.

With grammar accessing and combining lexical concepts, a new form of *propositional*, or in the terminology of the un-Cartesian hypothesis *grammatical*, meaning appears – the kind of meaning that arises from grammatical structuring of lexical-semantic information when the level of complexity of full sentences is reached. The existence and significance of this form of meaning has long been recognised, particularly in philosophy, but its potential origin in the meaning-giving function of grammar has not. Grammar, according to the un-Cartesian proposal, is what enables the “world as known”, the world as it appears in the format of propositional knowledge; it is what, as it were, forces us to think in a propositional format.

But now for the first time thought becomes subject to error. Sentences, unlike single words or phrases, are uniquely true or false: “man” cannot be true or false, nor can “the man” or “killed Bill”. However, “The man killed Bill”, uttered in context, can be.

One obvious advantage of the un-Cartesian hypothesis is its parsimony: there is no longer any need to postulate a separate LOT. Also speaking directly to the hypothesis is the fact that there is very little in the philosophical literature to suggest that, once words exist, we can actually distinguish between the notions of “word” and “concept”.^6^ (For further the evidence supporting the un-Cartesian hypothesis see Hinzen, 2014; Hinzen & Sheehan, 2013.)

## Applying the un-Cartesian hypothesis to delusions

Consider three specific examples of propositional delusions:

1. I am Jesus.
2. I have a wine glass in my stomach.
3. The Mafia is trying to kill me.

These statements do not immediately betray an abnormality of language. In particular, there is no anomaly at the *lexical* level: the words themselves are not being used in an unusual or novel way – in the first example the patient is talking about the same person as we do when we refer to Jesus, and the meanings of wine glass, stomach, the Mafia and killing are likewise entirely conventional. Nor does there seem to be any obvious violation of so-called selectional requirements, the constraints on how the individual parts of sentences can be coupled – we are not dealing with the kind of statements uttered by patients with formal thought disorder (the incoherent speech of schizophrenia) such as “The trains broke and the pond fell in the front doorway” (Oh, McCarthy, & McKenna, 2002), where the lexical features of ponds do not allow them to fall in doorways. Rather, (1)–(3) are pathological by virtue of, and only of, the way in which different sentence parts are combined at the level of *propositional or grammatical meaning*, where we see referential forms of meaning transpiring within specific grammatical configurations.

In (1), we see a sentence containing a subject picking out the speaker in the 1st Person, and a predicate in which we find a 3rd Person reference to a person, Jesus. In this case what makes the sentence un-propositional is that the *1*st *Person identity* of the speaker is fixed by reference to *a grammatical 3*rd *Person*. In health, though, no person referring to himself in the grammatical 1st Person will ever have a 3rd Person description as his or her identifying criterion. It is of course true that all of us fall under various 3rd Person descriptions: we are called by a certain name, are 6 ft tall, men or women, fathers, teachers, famous or ordinary; and indeed, we cherish some of our 3rd Person descriptions more than others. Yet whatever descriptions we pick, they are descriptions from a second or third person’s perspective, involving properties that, if they apply to us, apply to us for everyone. Our *1*st *Person identity*, however, is independent of all of them, and if any of these descriptions turn out not in the end to apply to us, the word “I” still refers to the same person, rather than a different one. The simplest way of putting this concept is to say that, as the word “I” functions in language, a person identified in the 1st Person can have various 3rd Personal properties, but it cannot *be* any of these. “I”, in essence, is always referential and it cannot be a property. It does not classify anything as belonging to a particular class, like all common nouns do.

It is because of this that identity sentences like (1) are restricted to those where both nominals are in the grammatical *3*rd *Person*, as seen in (4) and (5):

(4) Dr. Jekyll is Mr. Hyde.
(5) Superman is Clark Kent.

By contrast to these, it is clear that (1) is an *impossible* identity. It could only be uttered by a healthy speaker either non-literally (e.g., as a joke or metaphor) or, say, if I were a professional actor and was referring to a role I was playing in a historical movie that my interlocutor and I are watching. But in that case, the sentence is *not* an identity sentence of the kind it is in the pathological case: it would mean that I play the Jesus role – that is, I fall under a particular description, which is relevantly associated with another person, who is not me. Just conceivably, in the manner of a Dan Brown novel, I could perhaps carry Jesus’s genes, but in this case I still would not be him, that is, another person than I am. For the same reason, a person such as the Tokyo bomber, Shoko Asahara, who declared himself to be Jesus’s second coming, was not Jesus in the way a deluded patient states he is. (In fact, as we have noted earlier, a deluded patient would be unlikely to elaborate on or qualify such a belief in any meaningful way.)

In short, the deluded patient is doing something stronger than merely saying that he plays the role of Jesus, that he is a biological re-incarnation of Jesus due to the use of advanced technology, or that he is a historical person’s second appearance. Reference and predication *collapse*: What, in conjunction with a referential subject in the 1st Person, *could* in health only function as a 3rd Person predicate describing the speaker as being called by a certain name or playing a certain role, *becomes* a specification of his 1st Person *identity*. There are now *two* referential expressions, which, however, because they are in different grammatical Persons, cannot be the same; yet they are pathologically equated.^7^

In a delusion like (1) we have argued that a specific grammatical rule (in the un-Cartesian sense) is broken. In (2) there is again a violation of propositional or grammatical meaning, but this time it is more flagrant. According to standard textbook accounts of the philosophy of language (e.g., Lycan, 2008), it is integral to the concept of propositions that they carry information about the world; if they are true, they are true independently of my thinking them, and they still depict the world as being a certain way if they are false. However, there are very few circumstances where (2) could actually carry information about the world, or in other words where such a predicate as “in my stomach” could apply to the subject “a wine glass”. With sufficient ingenuity, a viable scenario might possibly be constructed – a freak accident, perhaps – but, as in the Jesus case only more so, the patient is not obviously constructing any such scenario: given what we know about how delusions are justified there is little reason to believe that he or she has any kind of backstory in mind; stomach and wine glass are simply juxtaposed and presented as a viable proposition.

If (2) is a gross violation of propositionality, (3) is at the other extreme. Here, an entirely possible event is being asserted. Yet even here a subtle form of the same problem can be detected. This is because, in health, referencing as an inherent aspect of propositional meaning always takes place in a “frame” where objects and events are located in relation to speakers, hearers, and in the context of other statements that have taken place before – any statement always follows on from other statements, which the persons conversing have both witnessed and mutually know they have witnessed. This frame, however, is constructed out of the grammatical meaning of previous utterances; in fact, it depends completely on them. However, in (3) the proposition is uttered in the absence of such a frame; the proposition does not refer to events in the world, as referred to in previous assertions, but instead only to the thinking process of the patient itself. The objects and events putatively referred to never become 3rd person objects and events of a sort that both the speaker (the 1st Person) and the hearer (the 2nd Person) can refer to as objects and events independent of them both. The “triangular” grammatical frame in which referencing necessarily takes place – that where an “I” addressing a “you” places an object or event (the “it”) in the world – is broken.

In a sense, the earlier arguments can be reduced to a single one: failure of propositional or grammatical meaning is what imparts to delusions their impossible quality. A corollary of this conclusion might then be that the severity of the violation will determine how bizarre the delusion is judged to be by others. At one end of the spectrum are delusions like “the Mafia are persecuting me” and “I have an incurable disease”, which are claims that could be made by people who are not mentally ill, but which turn out, on closer inspection, to simply not carry the propositional meaning they would be expected to have in healthy individuals. As the violation becomes more blatant, the result is delusions that are characterised as bizarre, such as “the Royal Family are stealing my inventions”; this really means that there is no way for others to easily assign propositional meaning to the statement. At the most extreme end of the spectrum, the utterances remain formally propositional but have lost all reference to the world as known, with patients saying things like “I have two mothers and many different fathers” and “I have invented a machine that can run the solar system powered by milk.”

## Accounting for other features of delusions

Although normal propositions are by nature true or false, their truth is usually not guaranteed: mistakes are always possible, as are lies. Why then are delusions asserted with such a degree of truth value that they are considered fixed and incorrigible? We propose that this feature of delusions can be understood in terms of another closely related linguistic disturbance.

One way of looking at the grammatical arguments made earlier is that they express ideas in which arguments are wrongly embedded under relevant predicates (e.g., being Jesus or having a wine glass in one’s stomach). However, whole clauses can also be embedded as arguments; from a grammatical point of view, the process is the same. Thus, verb phrases (VPs), for example, [_VP_ kill [_NP_ the dog]], contain noun phrases (NPs) embedded in them as subordinated arguments. But a sentence (S) too can be embedded, for example as an argument under a negation (e.g., [_S_ it is not the case that [_S_ the earth is flat]]), leading to an instance of “recursion”: S occurs subordinated within S, that is, itself. Like negation, verbs like “thinks” or “believes” embed clauses as well (e.g., [_VP_ thinks that [_S_ the earth is flat]]). In [_S_ John thinks [_S_ it is not the case that [_S_ the earth is flat]]], one sentence, “the earth is flat”, is embedded in another, “it is not the case that the earth is flat”, which is in turn embedded in yet another, “John thinks it is not the case that the earth is flat”, which contains all of the foregoing as subordinated parts.

We propose that not only do delusions wrongly embed arguments under their predicates, but also that clauses (of type S) which express delusions characteristically do not appear embedded in others. Deluded patients do not say, “I think I am Jesus” or “I feel like I have a wine glass in my stomach” or “It seems as though I am being persecuted by the Mafia”.^8^ These are very different propositions, and it is evident that the difference in their content flows from the differences in their grammar, specifically whether the sentence expressing the delusional content is embedded or not.

Furthermore, not only do delusions seem not to occur embedded, it seems quite possible that they cannot so occur and still be delusions.^9^ To illustrate what we mean here, consider what appears to be a counter-example, the case where a patient says that he *believes* he is Jesus. Conviction is expressed, but at the same time there is embedding. Here, though, there are only two possible interpretations of what the individual is saying. One is that he simply thinks he is (i.e., he considers the possibility that he is) Jesus, in which case he is not deluded – he does not have a fixed, incorrigible belief. The other is that he believes he is Jesus for a reason. The problem with this alternative is that a deluded patient is unlikely to make such a statement because by definition there can be no adequate reason for a delusion – if the reason was adequate, the belief would not be classified as a delusion. Thus, an individual who has had the experience of the Holy Spirit entering his body or has heard the voice of God telling him he is His son might be tempted to conclude he is Jesus, but there would be a huge amount of other evidence that would need to set aside before he was convinced. Even if he did manage to persuade himself, it would still not be reasonable to remain so after visiting a priest or a psychiatrist who reassures him that such experiences can also be a symptom of illness. In short, maintaining that one is Jesus for a reason is tantamount to saying that one can be rational about it or talked out of it, and these are not features of delusional belief.^10^

This brief digression also hints at a broader possibility that failure of embedding may be at the heart of what makes delusions illogical and not properly justified. Dialogue about a belief requires embedding it under negation and in the context of another mind potentially denying it (“you think … , but I think to the contrary … ”). In order to justify a claim that I am Jesus, I need to grasp the possibility that I am not; in order to discuss an idea about having a wine glass in my stomach, I need to be able to accept that I might be wrong. Inability to *embed* thus acquires some of the characteristics of inability to *represent*. This apparent relationship is explored further later, in the section on points of contact with other theories.

## Can the un-Cartesian proposal account for referential delusions?

So far, everything we have said applies to propositional delusions, an important type certainly, and what most people understand by the term delusion. Referential delusions seem to differ, in that, although they are technically propositions – the patient asserts that what he is saying is true – they have the additional feature that personal significance is attached to events. Is it a coincidence that the same term, reference, crops up both in linguistics and in relation to delusions?

We suspect not. As noted earlier, the concept of reference is basic to language; we cannot but use language referentially. The moment we use language, we *refer* to objects and events in the world, which we make statements about. A patient with referential delusions thinks acts of reference are taking place when in fact they are not, and/or he misinterprets himself as an object of reference with respect to real acts of reference directed to other people. If reference is a uniquely linguistic phenomenon, we might also expect such disturbances when the tool with which we handle reference, that is, language, malfunctions. These thoughts are preliminary, but they suggest that referential delusions may perhaps be an instance of a problem on the side of comprehension that in other ways manifests in expression as propositional delusions.

## Points of contact with other theories

*The work of Crow:* Clearly, any claim that delusions can be understood linguistically fits into the broader context of this author’s proposal that schizophrenia is the price Homo Sapiens pays for developing language (Crow, 1997, 2008, 2010). Crow’s argument is wide ranging, drawing on genetic, neuroanatomical and evolutionary lines of evidence, but at his most linguistic (Crow, 2010) he proposes that schizophrenic symptoms such as thought withdrawal, broadcasting and insertion, and also auditory hallucinations, reflect a disturbance of the deictic frame, the space in which an “I” – the centre of the deictic space – thinks, speaks, and refers.

Of course, disorders of the possession of thought like thought insertion and withdrawal, and perhaps also verbal auditory hallucinations, lend themselves to explanations in terms of deictic failure or confusion. However, the link with what we are saying may run deeper: this is because, as argued by Hinzen and Sheehan (2013), grammar as analysed in un-Cartesian linguistics proves to be nothing more or less than a reference system in which a person identifying himself in the grammatical 1st Person refers, *for* a 2nd Person, *to* some 3rd or non-Person object (“the world”), viewed as objective or independent of both the 1st and 2nd Person.

*Meta-representation:* Frith’s (1992) theory, in its full form rather than the narrow application to delusions referenced earlier, argues that the symptoms of schizophrenia involve a failure of representation. First-order representations are propositions (his term) about reality. Second-order representations are propositions concerning mental states, such as desires, pretence, and also beliefs (including others’ beliefs, or theory of mind). For example, “It is raining” is a first-order representation of the physical world, whereas “She believes it is raining” is a representation of a representation and so is a second-order representation or meta-representation. According to Frith, persecutory and referential delusions are due to a failure to properly represent what others are thinking. In contrast, “alien control” symptoms such as delusions of control and thought insertion can be understood as a failure to represent one’s own mental states. Frith has also attempted to account for auditory hallucinations using a more complicated argument along the same lines.

As we noted earlier, however, whereas the distinction between first- and second-order representations is a psychological one, in many ways it is homologous to the concept of embedding in language. After all, it is a linguistic feature, embedding one propositional unit in another, subordinating it, that distinguishes the one kind of representation from the other. In fact, with grammar at our disposal, particularly the broadened concept of grammar envisioned by the un-Cartesian hypothesis, it is not clear why a novel mechanism of “secondary” or “meta”-representation has to be posited at all: it comes, as it were, for free in a theory that proposes that all cognitive activity is linguistic and grammar structures thought. In this connection, some authors already propose that the whole concept of theory of mind may not be distinguishable from language, that is, from thinking with language or using particular linguistic forms for purposes of reasoning. This would apply to theory of mind in its full and “explicit” form (San Juan & Astington, 1995), which is strongly correlated with the mastery of clausal embedding, in both normal grammatical development (DeVilliers, 2007; San Juan & Astington, 1995) and atypical populations (DeVilliers & DeVilliers, 2012; Durrleman & Franck, 2015; Paynter & Peterson, 2010).

*Monothematic delusions in neurological disease:* So far, we have concerned ourselves exclusively with the kind of delusions seen in schizophrenia and other psychiatric disorders. However, patients with neurological disease also sometimes develop delusions which are present in the absence of any other psychopathology (i.e., they are not suffering from a superimposed psychotic disorder). Such “monothematic” delusions have been the subject of an important body of research that has attempted to understand their basis in terms of how brain damage can result in cognitive changes beyond simple neuropsychological deficits – the so-called cognitive neuropsychological approach or cognitive neuropsychiatry (Halligan & David, 2001).

By far the most studied example of this type of delusion is the Capgras syndrome, the belief that one’s partner and/or other family members have been replaced by doubles; this is well-documented as developing after a stroke or head injury and in the setting of several other neurological disorders (Edelstyn & Oyebode, 1999). Ellis and Young (1990) suggested that the Capgras delusion might be understandable as the mirror-image of the neurological syndrome of prosopagnosia, where patients become unable to recognise familiar people but often still show evidence of covert response to them, for example, as measured by autonomic arousal to a photograph of the person. In the Capgras patient, the argument goes, the cognitive machinery necessary for conscious recognition of familiar people remains intact but the parallel system that provides a jolt of emotional recognition when they are encountered is damaged. This then gives the patient a compelling feeling that a person or people who are close to him or her are somehow different or changed.

The challenge for cognitive neuropsychological theories is to explain why the Capgras patient does not immediately reject the explanation that the person must have been replaced, as patients with equally compelling abnormal experiences such as phantom limbs or tinnitus manage to do without difficulty. Clearly, an additional process of some kind needs to be invoked. After careful consideration over several years, and taking into account theories of neurological phenomena like confabulation, Coltheart and co-workers (Coltheart, 2005; Coltheart, Langdon, & McKay, 2011; Langdon & Coltheart, 2000; Turner & Coltheart, 2010) have concluded that this “second factor” must involve failure in a brain-based cognitive system that rapidly and automatically checks beliefs for their veridicality. The relevant process may be one of attaching a “feeling of rightness” to ideas that are judged to be consistent with the individual’s knowledge about the world as they enter conscious awareness; or alternatively it could be that ideas that are considered unusual for one reason or another are tagged with a label of “important, need careful checking”.

Coltheart and co-workers’ second factor seems not at all dissimilar to what we propose goes wrong linguisitically in the case of delusions that are not completely impossible, such as a belief that one is being persecuted by the Mafia. In these cases we argued that the propositional abnormality takes the form of a failure to place an assertion within a referential linguistic frame which is constructed out of the grammatical meaning of previous utterances. This process, it can also be noted, is by its very nature rapid and automatic – after all, it is carried out as a matter of course whenever people talk to each other. It is true that what Coltheart and co-workers envisage is conceptualised in terms of knowledge about the world, whereas referential linguistic frameworks are an intersubjective phenomenon taking place in the context of a dialogue, but the distance between these two concepts seems far from unbridgeable.

*Are delusions beliefs?* Recent years have been marked by debate over whether or not delusions can actually be considered to show the features of beliefs. The “anti-doxastic” position, that they do not, comes in various forms, from Berrios’s (1991) argument that delusions are empty speech acts, to Campbell’s (2001) contention that a different propositional meaning is expressed in a delusional statement compared to a normal one, to Currie’s (2000) suggestion that delusions are imaginations misidentified as beliefs. Counter-arguments have also been marshalled, notably by Bortolotti (2010) who, among other things, makes the point that examples of normal beliefs can be found that show the characteristics which cause delusions to fail tests of being beliefs.

By invoking a disturbance at the level of propositional meaning, the un-Cartesian position sidesteps the dichotomies around which the doxastic/anti-doxastic debate revolves. On the one hand, delusions in our account correspond to sentences that are asserted to be true, and so have all of the appearances of conveying a belief. On the other hand, they also differ from normal beliefs, by virtue of the fact that they lack normal propositional meaning as determined grammatically. But now there is no longer any need to say that this is because deluded patients make a complex meta-cognitive mistake in falsely believing that some thought they have is a belief when in fact it is an imagining. Rather, we can simply say that grammatical structure in language is a source of meaning of a particular kind, involving propositionality, and that this system malfunctions.

The un-Cartesian approach also avoids problems associated with Campbell’s (2001) proposal that a change in meaning has taken place. This is because it crucially distinguishes between changes of meaning at the level of the lexicon and those at the propositional level, and so has the option of saying that the former type of meaning is relatively intact, as it seems to be, whereas the use of language as a reference system is not.

## Concluding remarks

We have argued that the un-Cartesian approach allows what we term propositional delusions to be characterised linguistically, as a breakdown in the kind of meaning that words in combination acquire as a result of the operation of grammar. Taking such an approach seems to provide a plausible explanation of why delusions are not just false but impossible, ranging up to the bizarre and the fantastic. A variant of the same propositional-level abnormality, failure of clausal embedding, can also account for other key phenomenological features of delusions, namely their fixity, their failure to be justified by the patient and their lack of susceptibility to counter-argument. Although we have not developed the theme, it may also be that a corresponding set of linguistic features define referential delusions, perhaps acting on language comprehension rather than production.

Is this proposal a sterile exercise, one that simply redescribes the features of delusions using a linguistic vocabulary? We would argue that it is not, because being able to frame delusions linguistically brings them into line with the two other main classes of “positive” schizophrenic symptom, formal thought disorder and hallucinations. Formal thought disorder self-evidently involves language in the sense that it takes the form of disordered speech; perhaps more importantly several forms of linguistic abnormality have been empirically demonstrated in patients who show the symptom (for a review see McKenna & Oh, 2005). Similarly, schizophrenic hallucinations predominantly (though not exclusively) take the form of hearing people talking, and so any explanation will have to implicate language at some level, and current theorising recognises this (e.g., see Ford et al., 2014). It seems entirely possible that different aspects of linguistic representation could matter to symptoms in different ways – auditory verbal hallucinations might be a problem related to speech perception, whereas delusions relate to content, and formal thought disorder to language production (see Hinzen & Rosselló, 2015).

Of course, being able to formulate delusions in linguistic terms does not mean that they are thereby explained. A gulf remains to be bridged in identifying what biological or other processes could malfunction so as to result in impossible grammatical meaning and failure of embedding. Could these processes be neurochemical in nature, perhaps affecting the dopamine system, given that this is implicated in schizophrenia and that, as a positive symptom, delusions often respond well to antipsychotic drug treatment? On the face of it this possibility seems ruled out: among other things dopamine mediates reinforcement-based learning, and ever since Chomsky’s famous critique of Skinner, the idea that this type of learning could play a role in language has been anathema to linguists (e.g., see Harris, 1993). Nevertheless, whereas the idea of pathological neurochemical alterations directly influencing brain systems involved in grammar seems unlikely, the operative word here might be directly. Clearly, reinforcement-based learning is a fundamental requirement for the kinds of cognitive processes that, according to the un-Cartesian hypothesis, precede language. Ultimately, this leads to the formation of prelinguistic semantic structures, which define the world as it is experienced without language, and which can be highly complex in humans. This being so, it seems not impossible that delusions might somehow represent a later, indirect, linguistic manifestation of a pathological process that primarily takes place at a non-linguistic level.

This line of reasoning brings the un-Cartesian approach to delusions into contact with a final major theory of delusions, Kapur’s (2003) aberrant salience proposal. This maintains that dopaminergic hyperactivity, acting via the basic reward mechanism of salience attribution, causes neutral stimuli to acquire inappropriate motivational and reinforcing value. This in turn manifests itself as the subjective experience of neutral environmental events seeming significant, providing an immediate, plausible – and, one is tempted to say, prelinguistic – basis for referential delusions. Kapur’s explanation for propositional delusions is that they are the result of conscious efforts to make sense of such an altered experiential landscape; however, he does not offer any precise mechanism as to how this might come about. The un-Cartesian proposal might be able to step in here. For example, if it is assumed that prelinguistic experience is propositionalised on an ongoing basis as we make sense of new experiences, the potential exists for a pathological dynamic developing between the world as it is experienced prelinguistically, which is structured by reinforcement-based learning, and the world as it is known, which is articulated by language.

As a final note, it may be worth briefly revisiting the Jaspers’ (1959) view about the essential nature of delusions, that they are characterised by the sudden intrusive seeing of meaning. One way of looking at the approach to delusions we have developed in this paper is that it offers a way of applying Jaspers’ concept of abnormal meaning to not only referential delusions, where the argument is straightforward, but to propositional delusions as well – as disturbed propositional or grammatical meaning.

## Notes

1 We recognise that this terminology is not perfect – referential delusions are technically also propositional in the sense that the patient asserts that what is happening around him/her is true. However, it is difficult to find a better word to capture the distinction.

2 Not to be confused with the school of philosophy of the same name, although as Walker (1991) notes there are some links.

3 In his later writings, Chomsky (1995) articulated the view that crucial to the implementation of language in the brain is an “interface” with non-linguistic thought. This position maintains the divide in question. Very recently, however, Chomsky (2007) has adopted a position closer to the un-Cartesian hypothesis, according to which “I-language” (language as processed internally to the brain) and human-specific thought are in fact the same mental process, with an independent LOT being unnecessary.

4 This creativity, yet appropriateness, of our responses to given experiences is most directly manifested in ordinary language use. Except in the most formulaic of utterances, what we say is typically “new” rather than a selection from a finite set of pre-fabricated responses produced to a stimulus. In his debate with Skinner, Chomsky (1959) illustrated this point with reference to the utterance “Dutch” being made in response to a painting seen on a wall. For Skinner this would be a response under the control of extremely subtle properties of a stimulus, in this case a painting. Clearly, however, any of the following responses would have been equally appropriate, along with a potential infinity of others: “Clashes with the wallpaper.”; “I thought you liked abstract work.”; “Never saw it before.”; “Tilted.”; “Hanging too low.”; “Beautiful.” Describing all of these as stimulus-controlled would be circular, as only the response identifies the putative stimulus.

5 Richer, in particular, from a conception of grammar as formal “syntax”, which is defined through a methodological abstraction from the content properties of grammar.

6 Thus, a word viewed as a lexeme is only the lexeme it is if viewed together with its meaning. A word qua lexeme therefore uniquely identifies a concept, and any known concept uniquely identifies a lexeme. As proponents of pre-linguistic “concepts” themselves stress, moreover, these function differently from concepts that are lexemes, and they are more continuous with perception, as the present account predicts (e.g., Carey, 2009). Where concepts in the sense of lexemes *are* known to exist, they are in turn used referentially, which requires grammar. In contrast, in non-linguistic species, in whose communications there is no grammar in any human sense (including the “language-trained apes”, on which see Tomasello, 2008), reference takes a different format (Fitch, 2010).

7 This misidentification of a person in the 1st Person is crucially different from misidentifying or failing to identify itself in the 3rd Person. Thus, when I am amnesic I might not realise that I am indeed the person that I and others refer to as “Mr Smith”. In this case, I do not know who I am, but in that very question “Who am I?”, “I” picks out nothing but me, and 1st Person reference is crucially *intact*, even if I don’t know, for some 3rd Person descriptions, whether or not they apply to me, as identified in the 1st Person. (1) is a crucially different case, which is not possible with 1st Person reference in mental health: I mis-identify myself, in the 1st Person, as someone else.

8 We recognise that delusions are not always firmly held. However, so-called partial delusions (Wing et al., 1974) tend to occur in certain circumstances only, for example, at the beginning of illness or when fixed delusions fade as a result of treatment. We therefore feel it is reasonable to treat these as special cases.

9 We do not mean to argue that deluded patients are generally unable to embed clauses. This is obviously not the case, since they can express themselves normally on ordinary topics.

10 Interestingly, the patient who says, “I know I am Jesus” can be considered to be an exception that proves the above rule. Unlike “believe”, the verb “know” is a factive verb which introduces a presupposition that the proposition expressed by its clausal complement is true. Does this mean that what is decisive is not embedding but the meaning of the embedding verb? No, because although both “think” and “know” both clausal complements, their grammar is not the same: factive verbs have a distinctive grammar, which correlates with their different semantics (see e.g., Sheehan & Hinzen, 2011). Intuitively speaking (and simplifying), the difference is that factive embedded clauses are not truly embedded, functioning more independently.
